# Major anxiety disorders in Iran: prevalence, sociodemographic correlates and service utilization

**DOI:** 10.1186/s12888-018-1828-2

**Published:** 2018-08-20

**Authors:** Ahmad Hajebi, Seyed Abbas Motevalian, Afarin Rahimi-Movaghar, Vandad Sharifi, Masoumeh Amin-Esmaeili, Reza Radgoodarzi, Mitra Hefazi

**Affiliations:** 1 Research Center for Addiction and Risky Behavior (ReCARB), Iran University of Medical Sciences, Tehran, Iran; 2 Department of Psychiatry, School of Medicine, Iran University of Medical Sciences, Tehran, Iran; 3 Department of Epidemiology, School of Public Health, Iran University of Medical Sciences, Crossroads of Hemmat and Chamran Highways, Tehran, Iran; 4 Iranian National Center for Addiction Studies (INCAS), Tehran University of Medical Sciences, Tehran, Iran; 5 Psychiatry and Psychology Research Center, Tehran University of Medical Sciences, Tehran, Iran; 6 Department of Psychiatry, School of Medicine, Tehran University of Medical Sciences, Tehran, Iran

**Keywords:** Anxiety disorders, Generalized anxiety disorder, Obsessive-compulsive disorder, PTSD, Social phobia, Mental health services

## Abstract

**Background:**

It has been shown in the past two decades that anxiety disorders are the most common mental disorders in general population across the world. This study sought to assess the prevalence of major anxiety disorders, their sociodemographic correlates and mental health service utilization as part of the Iranian Mental Health Survey (IranMHS).

**Methods:**

A national household face-to-face survey was carried out on a representative sample of Iranian adults from January to June 2011 using Composite International Diagnostic Interview (CIDI 2.1). A total of 7886 subjects between 15 and 64 years who can understand Persian language were included. The 12-month prevalence of anxiety disorders according to Diagnostic and Statistical Manual of Mental Disorders, Fourth Edition (DSM-IV), their socio-demographic correlates, health service use and days out of role were measured in this study.

**Results:**

The 12-month prevalence of anxiety disorders (not including specific phobias) was 15.6%. The prevalence was 12.0% in males and 19.4% in females. The three most prevalent anxiety disorders were generalized anxiety disorder (5.2%), obsessive-compulsive disorder (5.1%) and social phobia (3.2%), respectively. Factors found to be significantly associated with anxiety disorders were: female gender (OR = 1.16, 95% CI: 1.09–1.23), middle (OR = 1.23, 95%CI: 1.01–1.50) or low (OR = 1.66, 95%CI: 1.31–2.10) socioeconomic status, unemployment (OR = 1.98, 95%CI: 1.49–2.62), and urban residence (OR = 1.31, 95%CI: 1.10–1.57). Comorbidity with non-anxiety disorders significantly increased service utilization. In all subgroups, service utilization was higher among females while the number of days out of role was higher among males.

**Conclusions:**

Anxiety disorders are common conditions with a higher prevalence among the female gender, unemployed individuals, and people with low socioeconomic conditions living in urban areas. Comorbidity of anxiety disorders with other psychological disorders aggravates the disability and significantly increases the number of days out of role.

## Background

Anxiety disorders are a group of psychological conditions characterized by an intense sense of anxiety and fear. Patients are mainly anxious about the future and their fear is a reaction to the current events. Several types of anxiety disorders exist, each with specific characteristics. However, they all share the signs and symptoms of anxiety. Anxiety disorders are often accompanied by other psychological conditions particularly major depressive disorder [1].

Epidemiologic studies conducted in the past two decades worldwide have shown that anxiety disorders are the most common mental disorders in general population [2–4]. The 12-month prevalence of anxiety disorders in different regions ranged from 6.4% in European nations [2] to 14.4 and 18.1% in Australia [3] and United States [4], respectively. Most of the anxiety disorders are more prevalent among women [5] and in developed compared to developing countries [6].

Epidemiologic studies have revealed that a significant percentage of Iranians are afflicted with mental disorders and psychological conditions. Study of the burden of diseases in Iran indicated that mental disorders ranked second after unintentional injuries in terms of burden of disease in Iran, comprising 16% of the overall burden of diseases [7]. In another study, the lifelong prevalence of psychological disorders was 10.8% in Iran, and anxiety disorders with a prevalence of 8.4% were reported to be the most common condition [8].

The Iranian Mental Health Survey (IranMHS) was carried out to estimate the prevalence, severity, service utilization pattern and costs of mental disorders. It has been the first study to assess the prevalence of psychological disorders in the national scale using the 12-month version of the Composite International Diagnostic Interview (CIDI 2.1) [9]. The current study aimed to assess the 12-month prevalence and severity of different types of anxiety disorders and their sociodemographic correlates in Iran.

## Methods

### Sample and study setting

The IranMHS was a national household survey with face-to-face interview carried out on a representative sample of Iranian adults aged 15–64 years. The exclusion criteria were: a) not understanding Persian language (4.8% of eligible individuals), b) not to be able to respond because of any severe illness (0.7%), c) not accessible at household during the study period (3.7%). The detailed methodology of the study has already been published [10]. A three-stage random sampling scheme was applied. In the first stage, 1525 blocks were chosen proportional to size of the population of each province based on the most recent national census. In the second stage, six households in each block were chosen by systematic random sampling and in the third stage, one member of each household was selected using the Kish grid tables. The study was carried out from January to June 2011 with a sample size of 7886 individuals. Among the 9150 households identified by interviewers; 506 (5.5%), 230 (2.5%), 329 (3.6%) and 98 (1.1%) did not participate in the study; respectively because of household refusal, household unavailability after at least 3 in-person contact, individual refusal and individual unavailability after 3 in-person contacts. Besides this, 17 (0.2%) respondents were found ineligible during interview and 84 (0.9%) questionnaires were eliminated because of their low quality. So, finally 7886 individuals out of 9150 households successfully participated in the study and the response rate was 86.2%.

### Variables and instruments

To assess the prevalence of anxiety disorders, Composite International Diagnostic Interview (CIDI2.1) was applied. This tool is a comprehensive, standard interview designed by the World Health Organization (WHO) to assess the 12-month prevalence of psychological disorders according to Diagnostic and Statistical Manual of Mental Disorders, Fourth Edition (DSM-IV). The anxiety disorders studied in IranMHS included: panic disorders (with and without agoraphobia), agoraphobia without panic disorder, Obsessive-Compulsive Disorder (OCD), social phobia, Generalized Anxiety Disorder (GAD) and Post-Traumatic Stress Disorder (PTSD). Sheehan Disability Scale (SDS) was used to assess the disability associated with psychiatric disorders [11]. Persian version of SDS, similar to its English version, has adequate validity and reliability [12]. We developed a service use questionnaire and evaluated its psychometric properties in the preliminary phase of the Iran-MHS as described elsewhere [10]. Socioeconomic status of participants was assessed using a household asset questionnaire. Principal component analysis was performed to estimate a socioeconomic score for each of the participants.

### Interviewer training, quality control, ethics and informed consent

Interviewers were 232 professionals with a university degree in psychology. Training consisted of a 56-h workshop of instruction and practice sessions that took place in 7 consecutive days in Tehran. Each Interviewer also took part in at least 2 pilot interviews with real subjects before the main phase of the study; and received appropriate feedback from experienced interviewers. In each province, a local executive manager and a supervisor (a member of research team) were assigned to ensure the quality of the procedures. The feasibility and acceptability of the interviews and the study limitations were assessed in a pilot study in selected urban and rural areas of Tehran and Khoozestan province. All interviews were conducted face-to-face at subjects’ place of residence. We made our best efforts to make sure that all interviews were conducted in privacy. All completed questionnaires were reviewed and edited by the local executive manager. A third of all questionnaires were double-checked by a supervisor. Errors or inconsistencies were returned to the interviewers for resolution. This involved re-interview with the participants in some cases.

### Sampling weights and statistical analysis

Sampling weights were calculated so that each respondent can be inflated to represent other individuals in Iran. The consolidated weights (w) were the joint product of inverse probability of unit selection into the sample (w1), non-response weights (w2) and post-stratification weights (w3): w = w1*w2*w3.

To calculate the post-stratification weights (w3), the proportion of subjects in each stratum in National Census 2006 was divided by the proportion of the same group in the sample. Based on 5-year age groups, sex and urban\rural place of residence in each of 31 country provinces, 1240 post-stratification weights were generated.

The sample sociodemographic structure was slightly different with national census data in terms of over-presentation of women, rural residents, homemakers and married individuals. However, after applying weighting procedures and doing complex sample analysis; the weighted proportions of different sociodemographic groups in the sample were found very similar to the census structure. The details are available in study protocol paper [10].

All statistical analyses including point prevalence estimates and their confidence interval were carried out using complex sample analysis procedures for taking into account the multi-stage sampling method and sampling weights. Data were analyzed using STATA/SE 10.0 software. Logistic regression (taking into account the complex sample structure) was applied to assess the association between independent variables and the study outcomes.

## Results

The overall prevalence of anxiety disorders in the past 12 months in the national MHS was found to be 15.6%. This rate was 12% in males and 19.4% in females. Higher prevalence of anxiety disorders in females compared to males was found for all types of anxiety disorders (Table 1). The three most common anxiety disorders in the study population were generalized anxiety disorder (5.2%), obsessive-compulsive disorder (5.1%) and social phobia (3.2%).

**Table 1:** Twelve-month prevalence of anxiety disorders in IranMHS (*n* = 7886)

Type of disorder	*n*	Total % (95%CI)	Female % (95%CI)	Male % (95%CI)
Any anxiety disorder		15.6 (14.5–16.6)	19.4 (17.9–20.9)	12.0 (10.6–13.4)
Panic disorder with/ without agoraphobia	178	1.9 (1.6–2.3)	2.5 (2.0–3.0)	1.4 (0.9–1.8)
Agoraphobia without panic	118	1.5 (1.1–1.8)	2.0 (1.5–2.5)	0.9 (0.5–1.4)
Social phobia	274	3.2 (2.7–3.6)	4.1 (3.4–4.8)	2.3 (1.7–2.9)
Generalized anxiety disorder	427	5.2 (4.6–5.8)	5.9 (5.2–6.7)	4.5 (3.7–5.4)
Obsessive-compulsive disorder	408	5.1 (4.5–5.7)	6.8 (5.9–7.6)	3.4 (2.7–4.1)
Post-traumatic stress disorder	169	2.1 (1.7–2.4)	2.4 (1.9–3.0)	1.7 (1.2–2.3)

The sex-specific interval estimates for 12-month prevalence of any anxiety disorder by demographic factors including: age groups, marital status, education, occupation, place of residence and socioeconomic status are described in Table 2. The findings shows that the higher prevalence of anxiety disorders in women is more prominent in younger age groups and it almost disappears in the oldest age group. Similarly, the gender difference of anxiety disorders is observed in both currently married and never married groups but the prevalence is almost equal between men and women in previously married participants. Another interesting finding is the decreasing trend of anxiety disorders by increasing educational level in male participants. Among female subjects, prevalence of anxiety disorders is highest in the middle education groups.

**Table 2:** Twelve-month prevalence of any anxiety disorders by socio-demographic characteristics

Variables	*n*	Any anxiety disorder			
		Female (*n* = 4499)		Male(*n* = 3387)	
		%	95% CI	%	95% CI
Age groups					
15–19	998	20.1	16.0–24.2	11.0	7.7–14.4
20–29	2549	19.7	17.4–22.0	11.6	9.3–13.9
30–39	2200	19.2	16.7–21.8	12.8	10.3–15.2
40–49	1188	19.2	15.5–22.8	12.8	9.4–16.3
50–59	704	19.6	14.7–24.6	11.6	6.7–16.4
60–64	247	13.8	7.6–20.0	13.5	4.0–23.1
Marital status					
Never married	2025	18.9	15.9–21.8	11.9	9.7–14.2
Married	5527	19.5	17.8–21.2	11.8	10.2–13.5
Previously Married	332	20.3	14.6–25.9	18.3	5.2–31.3
Education					
Illiterate	646	17.7	13.6–21.9	19.3	9.9–28.7
Primary	1917	22.3	19.2–25.3	12.2	9.3–15
Secondary	1280	24.1	20.1–28.2	12.9	9.9–15.9
High school	2823	18.1	15.7–20.5	11.9	9.6–14.1
University	1208	16	12.8–19.1	10.5	7.5–13.4
Occupation					
Employed	2803	17.9	14–21.8	10.9	9.4–12.5
Student	937	16.3	12.5–20.2	9.2	6.1–12.4
Retired	166	16.3	6.7–25.9	11.5	4.9–18.1
Housekeeper	3241	19.6	17.8–21.3	NA	
Unemployed	737	29.4	22.5–36.3	20.5	15.9–25.2
Place of Residence					
Urban	4380	19.1	17.2–21.0	12.5	10.7–14.2
Rural	3506	20.1	17.8–22.4	10.8	8.8–12.8
Socio-economic status					
Low	2152	23.3	20.4–26.2	16.1	12.9–19.3
Moderate	3191	19.5	17.3–21.8	12	9.8–14.2
High	2330	16.5	14.1–19.0	9.6	7.6–11.6

The association of sociodemographic variables with any anxiety disorder and the four most common diagnoses (namely: GAD, OCD, social phobia and PTSD) were assessed by applying five separate multivariate logistic regression analyses whose results are summarized in Table 3. The findings showed that female sex (Adjusted Odds Ratio = 1.16), unemployment (AOR = 1.98), urban living (AOR = 1.31) and moderate (AOR = 1.23) or low (AOR = 1.49) socioeconomic status were significantly associated with higher prevalence of any anxiety disorders. Being married (AOR = 1.51), unemployment (AOR = 2.15), urban living (AOR = 1.77) and low socioeconomic status (AOR = 1.54) were significantly associated with GAD. Young age, female sex (AOR = 1.17), unemployment (AOR = 2.45) and urban living (AOR = 1.37) were significantly associated with OCD. Female sex (AOR = 1.19), retirement (AOR = 3.79) and moderate (AOR = 1.76) and low (AOR = 2.85) socioeconomic status were significantly associated with social phobia. The only variable showing a significant association with PTSD was being divorced or widowed (AOR = 2.68).

**Table 3:** Socio-demographic correlates of anxiety disorders, findings of multiple logistic regression analysis

Variables	Any anxiety disorder	GAD	OCD	Social Phobia	PTSD
	Adjusted OR (95% CI)	Adjusted OR (95% CI)	Adjusted OR (95% CI)	Adjusted OR (95% CI)	Adjusted OR (95% CI)
Age groups					
15–19	1.0	1.0	1.0	1.0	1.0
20–29	0.88(0.66–1.20)	1.41(0.81–2.47)	1.17(1.06–1.30)^a^	1.20(0.70–2.06)	0.77(0.36–1.67)
30–39	0.89(0.64–1.24)	1.43(0.77–2.64)	0.86(0.55–1.35)	1.73(0.92–3.28)	0.70(0.32–1.55)
40–49	0.88(0.61–1.27)	1.47(0.76–2.86)	0.77(0.46–1.28)	1.09(0.53–2.26)	1.02(0.44–2.34)
50–59	0.85(0.55–1.31)	1.98(0.98–3.99)	0.52(0.29–0.94)	0.48(0.18–1.27)	1.15(0.43–3.08)
60–64	0.71(0.36–1.36)	2.16(0.79–5.93)	0.12(0.03–0.57)	0.29(0.07–1.23)	0.25(0.05–1.33)
Gender					
Male	1.0	1.0	1.0	1.0	1.0
Female	1.16(1.09–1.23)^a^	1.06(0.97–1.16)	1.17(1.06–1.30)^a^	1.19(1.08–1.32)^a^	1.11(0.95–1.28)
Marital status					
Never married	1.0	1.0	1.0	1.0	1.0
Married	1.08(0.85–1.36)	1.51(1.06–2.16)^a^	0.97(0.68–1.39)	0.58(0.38–0.89)	1.66(0.92–3.01)
Previously Married	1.09(0.71–1.67)	1.31(0.69–2.53)	1.01(0.51–2.00)	0.87(0.41–1.85)	2.68(1.02–7.04)^a^
Education					
Illiterate	1.0	1.0	1.0	1.0	1.0
Primary	1.09(0.78–1.53)	1.56(0.94–2.59)	0.84(0.48–1.46)	0.74(0.39–1.40)	1.41(0.64–3.10)
Secondary	1.19(0.83–1.71)	1.14(0.65–1.98)	0.87(0.50–1.52)	1.33(0.66–2.67)	1.34(0.57–3.17)
High school	1.01(0.71–1.44)	1.35(0.77–2.34)	0.63(0.36–1.12)	0.95(0.48–1.87)	1.16(0.50–2.69)
University	0.96(0.63–1.46)	1.59(0.82–3.10)	0.53(0.27–1.03)	1.17(0.52–2.63)	0.54(0.15–1.97)
Occupation					
Employed	1.0	1.0	1.0	1.0	1.0
Student	0.94(0.66–1.34)	0.82(0.45–1.51)	1.20(0.67–2.15)	1.22(0.66–2.26)	0.78(0.28–2.19)
Retired	1.16(0.64–2.11)	0.80(0.33–1.92)	1.54(.54–4.43)	3.79(1.19–12.1)^a^	0.27(0.03–2.28)
Housekeeper	1.07(0.82–1.40)	1.08(0.71–1.64)	1.29(0.80–2.07)	1.04(0.63–1.73)	0.81(0.42–1.58)
Unemployed	1.98(1.49–2.62)^a^	2.15(1.43–3.22)^a^	2.45(1.62–3.71)^a^	1.39(0.81–2.37)	1.44(0.71–2.89)
Place of Residence					
Rural	1.0	1.0	1.0	1.0	1.0
Urban	1.31(1.10–1.57)^a^	1.77(1.34–2.35)^a^	1.37(1.05–1.78)^a^	1.08(0.77–1.53)	0.96(0.66–1.41)
Socio-economic status					
High	1.0	1.0	1.0	1.0	1.0
Moderate	1.23(1.01–1.50)^a^	1.19(0.88–1.61)	1.13(0.81–1.57)	1.76(1.12–2.75)^a^	1.02(0.61–1.70)
Low	1.66(1.31–2.10)^a^	1.54(1.08–2.18)^a^	1.15(0.79–1.66)	2.85(1.71–4.74)^a^	1.37(0.82–1.41)

^a^highlights the statistically significant Odds Ratios

In Table 4; health service utilization, days out of role and severity of functional impairment are compared between four groups: [1] no disorder at all, [2] subjects with anxiety disorders only, [3] subjects with non-anxiety disorders only, and [4] subjects suffering from both anxiety and non-anxiety disorders. Comparison of service utilization showed that group 4 had the highest rate of utilization of services followed by groups 3 and 2. In other words, subjects with anxiety disorders alone had used health services less frequently than the other two groups of patients. Another important finding was that in all four groups, females had used services more frequently than males. However, this difference in some of the groups did not reach statistical significance. Both number of days out of role and severity of impairment were highest in group 4 followed by groups 3 and 2. None of these two were had statistically significant difference between men and women.

**Table 4:** Comparison of health service use and days out of role by anxiety and co-morbidity disorders

Variable		*n*	Health service use	Days out of roles	Severe impairment^a^
			% (95% CI)	Mean (95% CI)	% (95% CI)
No disorder	Male	2575	5.85 (4.70–7.00)	2.52 (1.93–3.11)	3.07 (2.19–3.96)
	Female	3033	12.85 (11.43–14.28)	2.36 (1.93–2.79)	3.50 (2.79–4.27)
Any anxiety disorder without comorbidity	Male	182	21.06 (14.54–27.58)	12.35 (7.44–17.27)	13.41 (7.48–19.35)
	Female	422	26.75 (21.86–31.64)	10.48 (6.07–14.90)	13.01 (8.65–17.37)
Any mental disorder not including anxiety disorders	Male	253	26.46 (20.21–32.71)	24.40 (16.83–31.97)	16.33 (10.96–21.70)
	Female	302	38.43 (31.54–45.32)	10.99 (8.16–13.81)	23.16 (17.70–28.61)
Any anxiety disorder comorbid with other mental disorders	Male	205	34.97 (27.19–42.75)	31.99 (23.57–40.42)	33.54 (25.92–41.16)
	Female	400	54.42 (48.52–60.31)	26.91 (21.60–32.16)	33.35 (27.82–38.87)

^a^At least two items of Sheehan with 70% or more disability

The prevalence of major depressive disorder (single or recurrent episode) among individuals who had any anxiety disorders was 42.9% (CI95%: 39.4–46.5%) while this prevalence proportion was 6.8% (CI95%: 6.0–7.6%) among those who did not have any anxiety disorders.

## Discussion

This study was the first to determine the prevalence of psychiatric disorders in the national level in Iran using a diagnostic tool like CIDI. Application of this tool allows for the comparison of results of this study with those conducted in other countries. In our study, the prevalence of anxiety disorders (15.6%) was higher than that of other psychiatric disorders. In the World Mental Health Survey, in all countries except for Ukraine, the prevalence of anxiety disorders was higher than that of other psychiatric disorders [13]. It is also noteworthy that the prevalence of anxiety disorders in all countries participating in the World Mental Health Survey except for the United States (18.2%) was lower than the rate in Iran [13]. Although some other diagnostic tools have been used in previous studies conducted in Iran, they reported higher prevalence of anxiety compared to other conditions, similar to the current investigation [8, 14, 15].

The current study results also showed that among anxiety disorders, the highest prevalence belonged to generalized anxiety disorder (5.2%) and obsessive-compulsive disorder (5.1%) while the lowest prevalence belonged to panic disorder with agoraphobia (0.5%). The results of our study regarding the prevalence of anxiety disorders are different from those of Mohammadi et al., [8]. In their study, the prevalence of phobia disorders (2.1%) was the highest followed by obsessive-compulsive disorder (1.8%) and panic disorder (1.5%). In a national survey in Australia [3], the most common anxiety disorder was PTSD (6.4%). In a study in Northern Ireland [16], the most common anxiety disorders were specific phobia (7.2%) and PTSD (5.1%), followed by social phobia (4.0%). In the United States [4], the highest prevalent anxiety disorders were specific phobia (8.7%), social phobia (6.8%), PTSD (3.5%) and GAD (3.1%), respectively. A study in Greece, showed that the prevalence (past seven days) of GAD, panic disorder and OCD were 4.1, 1.9 and 1.7%, respectively [17].

In the current study, assessment of the prevalence of anxiety disorders in the past 12 months prior to the interview showed that these disorders were more prevalent among women. This predilection for female gender was true for all types of anxiety disorders. Previous studies (both in Iran and other countries) yielded the same results as well [3, 4, 8]. The reasons behind the higher prevalence of anxiety disorders in females have yet to be clearly understood. Some previous studies have discussed the possible role of genetics in combination with environmental factors [18]. Others suggest the possible role of female sex hormones and related cycles in this regard [19, 20]. Imaging studies have shown that the anterior cingulate cortex in females with high fear response and harm avoidance is larger and more active than that in males with similar characteristics [21]. Although these findings have not been evaluated for all types of anxiety disorders, preliminary results show the higher susceptibility of women to anxiety disorders compared to men.

The results of the current study showed that the prevalence of anxiety disorders in females during the study period was higher in adolescents and young adults compared to other age groups. However, in males, this rate was reported to be higher among the middle-aged. Since the current study had a cross-sectional design, the obtained results can only indicate the higher prevalence of anxiety disorders in women at younger ages compared to men during the study period. Therefore, longitudinal studies are required to further scrutinize this topic.

This study revealed that aside from the significant role of female sex in prevalence of anxiety disorders, some other demographic factors could significantly affect the prevalence of these conditions. Low socioeconomic status, illiteracy, low level of education, unemployment and urban living are among the most important factors found in the current study. Evidence shows that people with lower socioeconomic status have poorer health indices [22]. Low level of education, low income, unhealthy lifestyle and occupational conditions and stress are among the socioeconomic factors often associated with poor health [23]. Evidence shows that illiteracy and low level of education marginalize people [24], decrease the access and utilization of available mental health services and interfere with some valuable goals in a community such as health promotion and access to social support and health care services [25, 26]. Low level of education is also correlated with poverty, unemployment and incarceration [26]. However, some studies have reported that if other factors such as social support are controlled, low level of education cannot serve as an independent risk factor for mental health problems [27]. Unemployment increases the risk of development and progression of psychiatric disorders and strong evidence is available in this regard for depression and anxiety disorders [28, 29]. Understanding of the fact that unemployment is a risk factor for occurrence of mental disorders plays a critical role in clinical and socioeconomic interventions. Although, this association might be the consequence of reverse causality. It means that unemployment might be the outcome of a psychiatric disorder, for example, Mojtabi et al. showed that having a mental illness can be accompanied by an increase in the chance of unemployment in the future [30]. Evidence shows that urban living has some advantages and disadvantages [31] and has been proposed as a risk factor for mental health disturbances [32, 33]. Air pollution, traffic noise, deprived districts, crime and poor social communication can adversely affect mental health [34]. Studies using magnetic resonance imaging have revealed increased activity of amygdala in urban residents when processing social stresses due to urban environment. Also, changed activity of perigenual anterior cingulate cortex has been found in subjects raised in urban areas. This region of the brain regulates the activity of amygdala, negative emotions and stresses [35]. However, it is difficult to recognize what specific characteristic of urban living/environment causes psychiatric disorders [36].

The current study revealed that presence of comorbidity in patients with anxiety disorders increased the percentage of service utilization, number of days out of role due to disorder and the percentage of individuals that developed severe disability. These results are in accord with the findings of studies showing that presence of a comorbidity in patients with anxiety disorders increased the severity of psychopathology, caused dysfunction and disability [37] and decreased the quality of life [38]. Presence of comorbidity can also affect the outcome of treatment of anxiety disorders [39] and is associated with an increase in signs and symptoms of anxiety disorders even after treatment [40]. The results of our study showed that both males and females with anxiety disorders used services more than subjects with no disorder, which is in accord with the findings of NCS-R study [41].

In our study, number of days out of role was higher in men compared to women, which is in contrast to the findings of a study conducted in the United States on the data provided by the collaborative psychiatric epidemiology studies (CPES) [5]. They showed that anxiety disorders in women were associated with higher number of days out of role. Possible reasons may be comorbidity of anxiety disorders with other conditions in women, higher social acceptance of sickness absence of women or a combination of both. One possible, yet important, reason of low number of days out of role in women in the national MHS in Iran is the attitude of a significant number of Iranian women towards being a housewife. A considerable percentage of women in our study were housewives. In most Iranian subcultures, being a housewife is not considered an occupation and therefore, it is not reported as one. Not being able to perform household tasks due to psychiatric disorders is not normally noticed and therefore, it is not reported by the subjects; this results in underestimation of data in this regard.

### Limitations

The major limitation of this study is not including specific phobias. In most population-based studies, specific phobias are found to be the most common type of anxiety disorders. So, the prevalence of any anxiety disorders we presented in this study is underestimated. In contrast to the World Mental Health Survey which had been administered in two steps [2], IranMHS was implemented in a single step. So the questionnaire was too long, and after exhaustive consultations, the study team decided to not include specific phobias in this study because of their less impact on the burden of psychiatric disorders [7].

Also, in this study it was not possible to translate CIDI to local languages; thus, those who were not able to understand Persian were excluded. This group of individuals comprised 4.8% of the target population and we have no information about the prevalence of anxiety disorders in this group. Thus, this may be considered as another limitation of this study. Considering the cross-sectional design of our study, we were not able to determine the precedence order of the factors such as socioeconomic status or unemployment and occurrence of anxiety disorders to analyze the role of influential factors. This is an inherent limitation of causal inference in all cross-sectional studies.

## Conclusion

The national MHS showed that anxiety disorders in Iran, similar to most countries participating in the World Mental Health Survey [2], were the most common type of psychiatric disorders. The prevalence of anxiety disorders (of all types) in females was 1.5 times the rate in males. These disorders were more commonly reported in adolescent and young-adult females and middle-aged males. Moreover, these disorders were more prevalent among the widowed, divorced, illiterate and unemployed female urban residents and male rural residents. Approximately, 50% of patients suffering from an anxiety disorder had at least one other psychiatric comorbidity. Based on the results, special attention must be paid to anxiety disorders because these conditions along with comorbidity significantly increase the severity of disability, number of days out of role and percentage of health service utilization. Increasing the quantitative capacity and promoting the quality of health care services provided, especially in the primary health care system, can enhance early detection and prompt treatment of these conditions and consequently decrease their prevalence and risk of chronicity.
